# Tissue-Resident Macrophage Development and Function

**DOI:** 10.3389/fcell.2020.617879

**Published:** 2021-01-08

**Authors:** Yinyu Wu, Karen K. Hirschi

**Affiliations:** ^1^Department of Medicine, Yale University School of Medicine, New Haven, CT, United States; ^2^Department of Genetics, Yale University School of Medicine, New Haven, CT, United States; ^3^Yale Cardiovascular Research Center, Yale University School of Medicine, New Haven, CT, United States; ^4^Vascular Biology and Therapeutics Program, Yale University School of Medicine, New Haven, CT, United States; ^5^Yale Stem Cell Center, Yale University School of Medicine, New Haven, CT, United States; ^6^Department of Cell Biology, Cardiovascular Research Center, University of Virginia, School of Medicine, Charlottesville, VA, United States

**Keywords:** primitive hematopoiesis, hemogenic endothelial cells, erythro-myeloid progenitors, definitive hematopoiesis, tissue-resident macrophages

## Abstract

Tissue-resident macrophages have been associated with important and diverse biological processes such as native immunity, tissue homeostasis and angiogenesis during development and postnatally. Thus, it is critical to understand the origins and functions of tissue-resident macrophages, as well as mechanisms underlying their regulation. It is now well accepted that murine macrophages are produced during three consecutive waves of hematopoietic development. The first wave of macrophage formation takes place during primitive hematopoiesis, which occurs in the yolk sac, and gives rise to primitive erythroid, megakaryocyte and macrophage progenitors. These “primitive” macrophage progenitors ultimately give rise to microglia in the adult brain. The second wave, which also occurs in the yolk sac, generates multipotent erythro-myeloid progenitors (EMP), which give rise to tissue-resident macrophages. Tissue-resident macrophages derived from EMP reside in diverse niches of different tissues except the brain, and demonstrate tissue-specific functions therein. The third wave of macrophages derives from hematopoietic stem cells (HSC) that are formed in the aorta-gonad-mesonephros (AGM) region of the embryo and migrate to, and colonize, the fetal liver. These HSC-derived macrophages are a long-lived pool that will last throughout adulthood. In this review, we discuss the developmental origins of tissue-resident macrophages, their molecular regulation in specific tissues, and their impact on embryonic development and postnatal homeostasis.

## Introduction

Tissue-resident macrophages are best known as immune sentinels that sense and respond to invading pathogens and challenging surroundings; they are also essential in tissue development, remodeling, and homeostasis (McGrath et al., 2015b). During embryonic development, macrophages are one of the first blood cell lineages to emerge. In mice, embryonic/fetal macrophages are known to originate during three distinct waves of hematopoiesis: primitive hematopoiesis, erythro-myeloid progenitor (EMP) generation, and definitive hematopoietic stem cell (HSC)-mediated hematopoiesis. Each wave differs but also overlaps temporally and spatially. Macrophages are then discretely positioned in the majority of developing organs (Davies et al., 2013).

As a consequence of diverse origins and the influence of tissue-specific microenvironments, tissue-resident macrophages are heterogeneous and exhibit tissue-specific functions during development and adulthood. Understanding the origins and development of tissue-resident macrophages will help us better understand tissue-macrophage heterogeneity, and provide insights into the fundamental differences between tissue-resident macrophages and adult monocyte-derived macrophages that reside in blood circulation (Davies et al., 2013). However, due to the complex nature of embryonic hematopoiesis, as well as the lack of specific phenotypic markers of different waves, it is very challenging to precisely delineate macrophage ontogeny. In this review, we will discuss what is known about the developmental origin of tissue-resident macrophages, their molecular regulation in specific tissues, and their functions during development and postnatally.

## Origins of Tissue-Resident Macrophages During Embryogenesis

### Primitive Hematopoiesis: The First Wave of Macrophage Emergence

**FIGURE 1:**
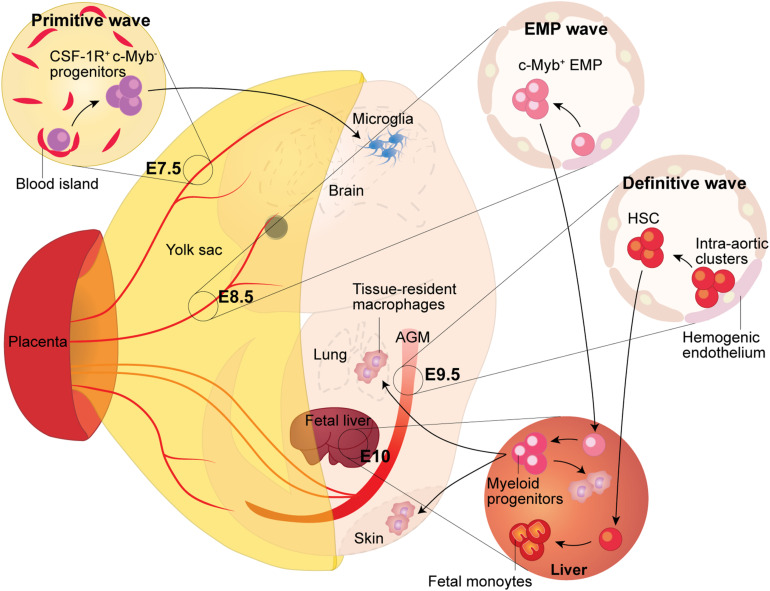
Fetal Macrophage Development. Murine macrophages originate from three successive waves of hematopoiesis. The first wave, termed primitive hematopoiesis, occurs in the blood islands of the extra-embryonic yolk sac at E7.5. It produces primitive erythroblasts and megakaryocytes, as well as CSF-1R^+^ c-Myb^-^ progenitors, which give rise to adult microglia in the brain. The second wave arises from the hemogenic endothelium formed at E8.5 in the yolk sac. Because the second wave generates the c-Myb^+^ hematopoietic progenitors named eythro-myeloid precursors (EMP), thus it is termed the EMP wave. The EMP either give rise to yolk sac macrophages locally or migrate into the fetal liver through the blood circulation, where they expand and differentiate into tissue-resident macrophages, which then migrate to, and colonize, different tissues. The third wave arises from the hemogenic endothelium in the aorta-gonad-mesonephros (AGM) region, which gives rise to fetal hematopoietic stem and progenitor cells beginning at E9.5. Subsequently, these precursors colonize the fetal liver where they establish definitive hematopoiesis and will eventually seed the bone marrow and lead to the generation of adult hematopoietic stem and progenitor cells.

In mice, primitive hematopoiesis begins around embryonic day (E)7.25 (Palis, 2016). The process occurs in distinct clusters of cells in the extra-embryonic yolk sac, called blood islands (Palis, 2016; Figure 1). Blood islands are composed of primitive hematopoietic cells in the center and sparse endothelial cells in the periphery (Hoeffel and Ginhoux, 2018). In the blood islands, primitive hematopoiesis generates unipotent myeloid progenitors that can only give rise to the macrophage lineage (Hoeffel and Ginhoux, 2018), as well as bipotent progenitors of erythrocytes and megakaryocytes (Tober et al., 2007). These progenitors remain in the blood islands until they are released during the onset of blood circulation around E8.0 and circulate throughout the embryo proper (Hoeffel and Ginhoux, 2018).

The first embryonic-derived macrophages are detected in the yolk sac at E9.0 (Naito et al., 1989; Takahashi et al., 1989). Although they are generated after initiation of both primitive hematopoiesis and EMP production, they are thought to be produced by the earlier primitive wave, considering the time needed for myeloid progenitors to self-expand and differentiate. Yolk sac-derived macrophages continue to proliferate and colonize the developing brain by E9.5 and the rest of the embryonic tissues by E12.5 until further diluted and replaced by macrophages generated from later waves (Schulz et al., 2012; Hoeffel et al., 2015; McGrath et al., 2015b; Palis, 2016).

Unlike macrophages that are generated during the two successive waves of hematopoiesis, macrophages originating during primitive hematopoiesis are generated directly from progenitors without going through a monocyte intermediate (Naito et al., 1989; Takahashi et al., 1989). Indeed, in terms of developmental timeline, the appearance of primitive macrophages is observed prior to monocytes in the mouse embryo, and when E8.0 mouse yolk sac progenitors are cultured *in vitro*, they do not produce monocyte precursors (Naito et al., 1989; Takahashi et al., 1989). More direct evidence that macrophages derive directly from yolk sac progenitors *in vivo* have come from recent fate mapping studies. Using inducible *Runx1* promoter-driven GFP reporter mice at specific time points in development, Ginhoux et al. (2010) demonstrated that microglia (tissue-resident macrophages in the adult CNS) originate directly from E7.5 yolk sac progenitors. Progenitors for microglia were further defined as the CD45^–^c-Kit^+^ population in the brain (Kierdorf et al., 2013). Using reporter mice labeled with inducible colony stimulating factor 1 receptor *(Csf1r)-Cre*, Gomez Perdiguero et al. (2015) also reported that tissue-resident macrophages are predominantly derived from yolk-sac progenitors that express CSF1R^+^ at E8.5. They also found that a large number of tissue-resident macrophages, including macrophages in liver, brain, epidermis and lung, originate via Tie-2-expressing cells before E10.5 (Gomez Perdiguero et al., 2015). Macrophages generated directly from these progenitors possess high proliferative potential, enabling them to expand and maintain their pools in the majority of tissues over time in the embryo (Takahashi et al., 1989; Palis, 2016). In contrast, the brain is the only tissue that maintains macrophages generated during primitive hematopoiesis throughout adulthood (Alliot et al., 1991; Alliot et al., 1999; Ginhoux et al., 2010).

Although it has been recognized for some time that macrophages are generated during primitive hematopoiesis, the underlying mechanisms that direct their development are still largely unknown. Nonetheless, it is well accepted that myelopoiesis during primitive hematopoiesis is not dependent on regulation by c-Myb, a transcription factor required for the development of HSC (Sumner et al., 2000; Schulz et al., 2012). Indeed, *c-Myb*^–/–^ mouse embryos exhibit depletion of most other myeloid cells at E16.5 except yolk sac macrophages (Schulz et al., 2012).

Since microglia are the only type of primitive macrophages that persist throughout adulthood, more studies have been focused on the mechanisms of microglia development. Kierdorf et al. (2013) found that hematopoietic transcription factors PU.1 (also known as Spi1) and Interferon regulatory factor-8 (IRF8) are required for the development of microglia. They also observed that primitive c-Kit^+^ precursors developed and matured into c-Kit^–^CD45^+^CX_3_CR1^+^ progenitor cells with downregulation of CD31 and concomitant upregulation of adult macrophage marker F4/80, as well as macrophage colony stimulating factor receptor (MCSF-R). The c-Kit^–^CD45^+^CX_3_CR1^+^ progenitor cells are proliferative, able to infiltrate the developing brain and subsequently develop into microglia.

Development of microglia also requires transforming growth factor β (TGF-β) signaling. Adult microglia cultured *in vitro* without the addition of TGF-β1 fail to express microglia signature genes and no microglia are detected in the brain of adult TGF-β1-deficient mice (Butovsky et al., 2014). Another study utilized mice expressing a *Vav1*-driven inducible Cre to delete *Tgfbr2* (gene encoding transforming growth factor β receptor 2) in hematopoietic cells. In this model, at E11.5, microglia were significantly reduced in the brain during development (Utz et al., 2020).

### Yolk Sac Hemogenic Endothelium Give Rise to EMP

The second wave of macrophage generation arise from the EMP in the yolk sac around E8.25 (Hoeffel et al., 2015; Figure 1). EMP are derived from hemogenic endothelium in the yolk sac vasculature, a transient, specialized vascular endothelium with definitive hematopoietic potential (Gritz and Hirschi, 2016). EMP are highly proliferative, expanding predominantly in the yolk sac. Although EMP are detected in the embryonic bloodstream through E12.5, they start to seed the developing fetal liver by E10.5 (Palis et al., 2001; Frame et al., 2014; McGrath et al., 2015b). In circulation-deficient mouse embryos, these macrophages are retained in the yolk sac, suggesting they require blood circulation to migrate to other embryonic tissues (Ginhoux et al., 2010; Hoeffel et al., 2012).

Erythro-myeloid progenitors in the fetal liver produce the first enucleated erythrocytes and give rise to bipotent granulocyte/macrophage progenitors and mast cells (Palis et al., 1999; Bertrand et al., 2005; McGrath et al., 2015b). Recently, McGrath et al. (2015a) revisited the lineage potential of the EMP and found they give rise to erythrocytes, mast cells, basophils, neutrophils and macrophages in culture. However, EMP are short-term progenitors; transplantation of E10.5 EMP into either normal or immune-compromised adult mouse recipients produce erythroid cells, with very limited myeloid cells or platelets (McGrath et al., 2015a). Due to lack of long-term potential in adults, the EMP wave of hematopoiesis is also called “transient definitive wave” (Hoeffel and Ginhoux, 2018).

Due to temporal and spatial overlap of EMP-mediated hematopoiesis with primitive and HSC-derived myelinogenesis, it is important to identify unique cell surface markers for EMP to better distinguish them from myeloid cells generated during the other two processes. McGrath et al. found that, in E9.5 mouse yolk sac, c-Kit^+^CD41^+^ EMP share markers with hemogenic endothelial cells and developing HSC (i.e., CD31, CD45, and CD61). However, EMP express CD16/32 and not Sca-1; thus, distinguishing them from HSC. In addition, although AA4.1 (also known as CD93) is highly expressed by fetal liver HSC, it is expressed by only 15% of E9.5 EMP; the majority of AA4.1^+^ cells in E9.5 yolk sacs are c-Kit^–^, vascular endothelial (VE)-cadherin^+^ endothelial cells (McGrath et al., 2015a). Hoeffel et al. (2015) further verified that c-Kit^+^CD41^+^CD16/32^+^CD93^–^ EMP can give rise to almost all tissue-resident macrophages found in adults via monocytic intermediates.

In other studies, using a fate mapping approach, CSF1R-expressing cells in the mouse embryo were found to give rise to tissue-resident macrophages in adult tissues (Schulz et al., 2012); however, the generation of EMP does not require CSF1R (Ginhoux et al., 2010; Hoeffel et al., 2012). In addition, toll-like receptor 2 (TLR2) was found to be essential for E8.5 c-Kit^+^ yolk sac EMP development and function, and can be used as a cell surface marker to distinguish them from cells generated during primitive hematopoiesis (Balounová et al., 2019).

The fact that *c-Myb*^–/–^ mouse fetal livers are devoid of the EMP-containing c-Kit^+^ population suggests that development of EMP may be c-Myb dependent (Schulz et al., 2012). Indeed, c-Myb is expressed in EMP in the E9.5 yolk sac, as well as in the fetal liver (Hoeffel et al., 2015). Like HSC development, EMP development requires Runx1. Runx1-deficient mouse embryos die with lack of fetal liver hematopoiesis around E12.5. Runx1-deficient mouse embryonic stem cells (ESC) demonstrate no colony-forming activity in culture. Moreover, injecting Runx1-deficient mouse ESC into blastocysts of wild type recipients reveals no myeloid lineage potential from the Runx1-deficient cells (Okuda et al., 1996). The binding partner of Runx1, core binding factor β (Cbfβ) is also indispensable in EMP development. In *Cbf*β^–/–^ mice, forced expression of Cbfβ driven by *Tie-2* promoter rescues the number of EMP in E12.5 fetal liver (Miller et al., 2002). The role of Runx1 and its binding partner in EMP development are also involved in the endothelial-to-hematopoietic transition (EHT) and HSC generation (Palis, 2016). However, unlike the HSC-associated EHT, development of EMP does not require blood flow or Notch1 (Hadland et al., 2004; Frame et al., 2016).

Several studies have also demonstrated that fetal monocytes contribute to adult macrophages in different tissues. In one study, Hoeffel et al. (2015) depleted yolk sac macrophages in mouse embryonic tissues, but left monocytes intact, by deleting CSF1R, which is essential to development and maturation of macrophages (Hamilton, 2008). They found that fetal monocytes differentiate into macrophages in the skin, lung, liver, kidney, and gut before birth. In homeostatic conditions, activation of *CSF1R* or *Runx1* promoter-driven reporters at E8.5-9.5 leads to labeling of E10.5 EMP. Reporter activation at E13.5 leads to labeling of non-brain fetal macrophages, fetal liver monocytes, granulocytes and adult fetal-derived macrophages except microglia. In other lineage tracing studies utilizing the promoter of *S100a4*, a gene actively expressed in monocytes, activating the reporter from E14.5 onward labels macrophages in all tissues except microglia (Hoeffel et al., 2015). Since no phenotypic marker is known to distinguish macrophages derived from distinct hematopoietic sources, these lineage-tracing studies delineating the origin of fetal monocyte-derived macrophages are highly dependent on the time window of the labeling and tracing studies, relative to the onset of distinct hematopoietic waves. Thus, the temporal overlap of different hematopoietic processes during embryonic development complicates these analyses.

Another issue is that HSC also produce fetal monocytes that generate a small number of resident macrophages in the perinatal period. Studies were done to try to delineate differences in the fates of fetal monocyte-derived macrophages generated from EMP-mediated vs. HSC-mediated waves of hematopoiesis. Guilliams et al. isolated CD45.1^+^ fetal macrophages and CD45.2^+^ monocytes from E17 lungs, and transferred them in a 1:1 ratio into CD45.1^+^CD45.2^+^ healthy recipient newborn mice. They found that, among recipient lung alveolar macrophages, significantly more are derived from fetal monocytes, indicating they are the main precursors for lung tissue-resident macrophages (Guilliams et al., 2013).

Collectively, these studies suggest that EMP give rise to most fetal-derived tissue-resident macrophages with the exception of brain microglia. Although fetal HSC-derived monocytes may also contribute to tissue-resident macrophages, their overall contribution is not clear.

### HSC Generate Macrophages During Definitive Hematopoiesis

HSC-dependent definitive hematopoiesis begins at approximately E9.5 in the mouse intra-embryonic AGM region (Medvinsky and Dzierzak, 1996; Figure 1), as well as in vitelline and umbilical arteries (Zovein et al., 2010). Like EMP, HSC are also derived from hemogenic endothelium, which undergoes EHT (Gritz and Hirschi, 2016). Despite the fact that they have the same cellular origin, regulation of HSC development differs significantly from the emergence of EMP (for detailed review, see Wu and Hirschi, 2020). HSC emerge in clusters budding from the endothelial lining of the aortic wall in the AGM region, and then migrate into the developing fetal liver, where they undergo maturation and massive self-expansion. By E16.5, the HSC migrate to and colonize the developing fetal bone marrow, where they remain throughout adulthood, and generate all needed blood cell lineages (Dzierzak and Speck, 2008; Coskun et al., 2014).

It was previously thought that circulating monocytes originated from bone marrow constitute the only precursors for all tissue-resident macrophages in the adult (van Furth et al., 1972). However, as mentioned above, this paradigm was challenged by recent findings that most tissue-resident macrophages have a yolk sac origin. Although adult monocytes can contribute to tissue-macrophage populations, HSC-derived tissue-resident macrophages only marginally replace the yolk-sac-derived macrophages in steady-state conditions in 1-year old mouse tissues and organs, notably the brain, liver and epidermis (Gomez Perdiguero et al., 2015).

Interestingly, EMP-derived macrophages can be replaced by HSC-derived macrophages in some tissues early in the postnatal period. For example, in mouse intestine, EMP-derived macrophages colonize the intestinal mucosa, but do not persist into adulthood. During the weaning period, they are diluted by tissue-resident macrophages that were generated by HSC-derived Sca-1^+^ monocytes. This process is dependent on the chemokine receptor CCR2 and commensal microbiota, and continues throughout adult life (Bain et al., 2014). A similar process was also observed in skin, spleen and heart, in which fetal monocyte-derived macrophages are replaced by adult monocyte-derived macrophages progressively over time (Tamoutounour et al., 2013; Molawi et al., 2014; Hoeffel et al., 2015). Importantly, yolk sac- and HSC-derived macrophages can coexist in the same tissue and maintain a balance in the steady state, except under inflammatory conditions, when large numbers of HSC-derived macrophages invade tissues, such as heart (Yap et al., 2019) and liver (Blériot et al., 2020). However, the contributions of the developmentally distinct macrophage populations to tissue homeostasis and inflammation remain to be investigated (Davies et al., 2013).

## Roles of Tissue-Resident Macrophages in Embryogenesis

### Angiogenesis and Neurogenesis

Tissue-resident macrophages have been reported to regulate neurogenesis. For example, microglia were found to modulate neuron outgrowth and positioning in a CX_3_CR1-dependent manner during murine brain development (Squarzoni et al., 2014). In addition, in the developing mouse testis, fetal macrophages derived during primitive hematopoiesis can mediate tissue vascularization (Defalco et al., 2014). Interestingly, a recent finding by Plein et al. suggests another relationship between blood and endothelial lineages. Using inducible *Csf1r* promoter-driven-Cre ROSA^*YFP*^ reporter mice, they found that circulating EMP contribute to endothelial cells lining blood vessels in multiple tissues, including the liver, brain, heart, lung, and yolk sac, and persist throughout adulthood. Unlike the classic differentiation of angioblasts to endothelium, these EMP are thought to revert to their initial endothelial fate and intersperse within existing vessels (Plein et al., 2018). However, other recent studies by Feng et al. (2020) argue that there is no evidence for EMP-derived vascular endothelial cells in the above-mentioned organs. Thus, the contribution of EMP to endothelial cells needs further investigation.

### Osteogenesis

Erythro-myeloid progenitors also give rise to the majority of osteoclasts in mouse neonatal bones. Using lineage tracing and single-cell RNA-sequencing techniques, Yahara et al. (2020) found that CX_3_CR1^+^ EMP-derived macrophages fuse with local HSC-derived macrophages to form multinucleated long-lasting osteoclasts, which contribute to postnatal bone remodeling in homeostatic and inflammatory conditions.

### Erythropoiesis

Macrophages in the blood islands play an important role in supporting erythropoiesis in both homeostatic and stress conditions. They have been shown to attach directly to mouse erythroblast cells and promote their proliferation in culture (Rhodes et al., 2008). Fetal liver-derived macrophages also adhere to mouse primitive erythroblasts and promote their enucleation (McGrath et al., 2008). A similar phenomenon was also observed in human placentas, where human primitive erythroblasts enucleate when in contact with macrophages (Van Handel et al., 2010). Other studies also show that erythroblastic island macrophages play an important role in the clearance of pyrenocytes in homeostatic conditions, and macrophage depletion significantly impairs erythropoietic recovery from hemolytic anemia, acute blood loss and myeloablation (Chow et al., 2013; Ramos et al., 2013).

### Clearance of Cell Debris

Another important role of macrophages in development is clearance of cell debris and maintenance of tissue homeostasis. For example, it has been reported that macrophages digest the apoptotic cells in the digits of the remodeling limb bud during mouse limb morphogenesis (Hopkinson-Woolley et al., 1994). Macrophages can also remove germ cells and somatic cells that fail to incorporate into cords during testis development (Defalco et al., 2014). Aging red blood cells are also found to be cleared by splenic macrophages (de Back et al., 2014).

## Postnatal Functions of Embryonic/Fetal-Derived Macrophages

One of the main functions of postnatal tissue-resident macrophages is to sense the perturbation of the microenvironment and induce inflammation. For example, in experimental inflammation conditions, depletion of resident macrophages results in diminished chemokine production, leading to a dramatic attenuation of neutrophil influx, which can be rescued by adoptive transfer of resident macrophages (Cailhier et al., 2005). However, due to the variable nature of the perturbation, the different types and distribution of the expressed recognition receptors, the exact role of tissue-resident macrophages in inflammation induction varies (Davies et al., 2013). Tissue-resident macrophages in postnatal tissue also function in repair processes and wound healing. In addition, microglia stimulate recruitment of anti-inflammatory macrophages from the periphery that promote resolution of tissue injury (Shechter et al., 2013).

## Summary

The successive waves of embryonic and fetal macrophage generation are complicated. To date, it is not yet understood how the diverse sources impact the function of tissue-resident macrophages. Thus, further investigation of the impact of hematopoietic origin, and the timing and sites of generation, tissue colonization and maturation are needed to better understand the diverse phenotypes and functions of tissue-resident macrophages. Indeed, recent advances in single-cell RNA-sequencing are helping to provide more comprehensive characterization of early macrophage development in both mouse and human embryogenesis (Bian et al., 2020; Yahara et al., 2020). In addition, local environment-specific signals are likely to act coordinately with the inherent ontogenetic factors to influence the phenotypes and behavior of tissue macrophages. Thus, defining the cell-autonomous and non-autonomous regulators of tissue-resident macrophages will be necessary to fully understand their functions and, ultimately, how to manipulate their behavior *in vivo*.

## Author Contributions

Both authors listed have made a substantial, direct and intellectual contribution to the work, and approved it for publication.

## Conflict of Interest

The authors declare that the research was conducted in the absence of any commercial or financial relationships that could be construed as a potential conflict of interest.
